# The Role of Dietary Habits in the Pathogenesis and Development of Inflammatory Bowel Disease: A Narrative Review

**DOI:** 10.34172/mejdd.2024.362

**Published:** 2024-01-31

**Authors:** Mitra Ahadi, Mohammad Reza Rouhbakhsh Zahmatkesh, Parisa Ebrahimi, Mina AkbariRad

**Affiliations:** ^1^Department of Internal Medicine, Faculty of Medicine, Mashhad University of Medical Science, Mashhad, Iran; ^2^Faculty of Medicine, Mashhad University of Medical Science, Mashhad, Iran

**Keywords:** Crohn’s disease, Diet, Dietary habits, Immune-mediated disease, Inflammatory bowel disease, Ulcerative colitis

## Abstract

Inflammatory bowel disease (IBD), including Crohn’s disease (CD) and ulcerative colitis (UC), is a chronic immune-mediated disease. The incidence of IBD is influenced by various genetic and environmental factors, with dietary habits gaining significant scientific interest. While the role of diet in the pathogenesis and development of IBD is still debated, recent studies have demonstrated its potential impact. However, conflicting findings exist regarding the efficacy of dietary interventions in the treatment and control of IBD. This review aimed to summarize the current understanding of the relationship between diet and IBD, highlighting the different perspectives and reasonings observed in recent studies. Overall, it has been shown that dietary habits play a role in the incidence of IBD, and adopting a controlled dietary approach may help manage the disease. Consequently, diet can be considered a predictive and prognostic factor in IBD.

## Introduction

 Inflammatory bowel disease (IBD), which encompasses Crohn’s disease (CD) and ulcerative colitis (UC), is a chronic immune-mediated condition characterized by periods of exacerbation and remission.^1,2^ While the exact etiology of IBD remains elusive, it is hypothesized that the disease arises from alterations in cell-wall contracture, increased cell-wall permeability, and immune reactions targeting the intestinal and extra-intestinal cell walls.^3^ The primary clinical manifestations of IBD include abdominal pain, rectal bleeding, and persistent diarrhea, while additional extra-intestinal signs and symptoms involving skin abnormalities and joint problems may also be present.^3^

 Recent studies have reported a wide range of IBD incidences. A survey conducted in 2012 revealed that the incidence of UC ranged from 0.6 to 505 per 100 000 individuals, while for CD, it ranged from 0.6 to 322 per 100 000 individuals.^4^ Notably, industrialized countries exhibit a higher prevalence of IBD than developing nations. The highest incidence rates of UC and CD are observed in the age range of 20 to 40 years, with no significant sex-based differences in disease susceptibility.^4^

 The contributing factors to IBD encompass genetic and environmental elements.^5^ The genetic component of the disease has been inferred from studies linking IBD with positive family histories among patients. Twin studies have further underscored the significant role of hereditary factors in IBD.^6^ Environmental factors, on the other hand, are primarily elucidated in terms of socioeconomic conditions such as smoking, cultural background, personal hygiene, and dietary habits.^7-9^ The severity of the disease is evaluated using a combination of clinical symptoms and laboratory data, mainly fecal calprotectin, although it has not been proven to show a more significant rise in patients with more severe disease.^10^

 The association between dietary habits and IBD has long been a prominent area of research. Malnutrition is a common consequence of IBD, particularly in individuals with CD. The combination of nutrient malabsorption and reduced oral intake has been demonstrated to contribute to malnutrition.^11^ Also, in patients with UC, significant deficiencies in zinc and selenium were reported compared with healthy individuals.^12^ Adequate nutritional support is crucial in preventing complications arising from nutrient deficiencies.^13^ Although limited information is available on the use of diet in managing and preventing IBD,^14^ the efficacy of dietary interventions for treating and controlling the disease remains a subject of debate.^15^ Nevertheless, several studies have indicated that IBD flares are not significantly correlated with any specific dietary habits.^16^ Given the rising incidence rates of IBD, there is a growing interest in preventive measures, with dietary intervention being one of the primary interventions under evaluation in this study.

## Materials and Methods

 A comprehensive search was conducted using databases such as Scopus, Google Scholar, MEDLINE, PubMed, and Science Direct. The search utilized keywords including “diet,” “inflammatory bowel disease,” “ulcerative colitis,” and “Crohn’s disease” to identify relevant studies involving dietary interventions in patients with IBD. The inclusion criteria encompassed English studies conducted on both humans and animals, published between 1953 and 2022, along with their associated references. The titles and abstracts of the identified studies were carefully reviewed to identify potentially relevant data.

 A total of 97 studies were explored during the search process. Among these, 80 studies were excluded due to their outdated publication dates or non-relevant methodologies. Ultimately, 17 studies were included in the review. These studies varied in terms of sample sizes and study methods, with the study designs comprising both prospective and retrospective approaches.

## The Role of Diet in the Pathogenesis of IBD

 It is widely acknowledged that diet plays a significant role in the pathogenesis of IBD. This influence is primarily mediated through its impact on the composition of the intestinal microbiome, intestinal integrity, and host immunity. Consumption of certain nutrients has been shown to trigger detrimental effects, leading to the aforementioned alterations.^17^ Specifically, studies have demonstrated that dietary habits characterized by high fat, high sugar, low fiber, high lactose, and gluten-enriched diets can have particularly destructive effects.^18^ Notably, changes in dietary habits and individual nutrient intakes have been implicated in the dysregulation of the gut microbiota, a condition referred to as “dysbiosis,” which is observed in various patients with IBD.^19,20^

## Western Diet

 A Western diet refers to a dietary pattern characterized by a high intake of fat and sugar and a low intake of fiber. This type of diet has been associated with gut dysbiosis, increased intestinal permeability, compromised intestinal integrity, heightened immune response, and activation of intestinal immune cells, thereby increasing the risk of developing IBD.^21,22^

 The mucous layer of the intestine serves as the initial site of interaction between food antigens and the immune system, triggering an immune response in the presence of these agents. The non-constitutive and irregular immune response to pathogens and food antigens can lead to changes in the innate immune system, activation of T cells, increased presence of B cells, and production of specific antibodies. Additionally, the production of pro-inflammatory mediators, along with the presence of B cells and antibodies, as well as an increase in the production of pro-inflammatory factors, all contribute to the pathogenesis of IBD.^23^

 In UC, inflammation is limited to the mucosal layer, which leads to superficial damage to the endothelial tissues of the colon.^24^ Additionally, in UC, a dysregulated immune reaction modifies the gut environment by augmenting the disproportionate secretion of compound pro-inflammatory factors. Therefore, the basic principle of the dysregulated immune system following consumption and the UC is proven in the study, which assessed the relationship between oral mebendazole and the mucus layer composition.^25^

 There is a growing body of evidence highlighting the impact of a Western diet on the gut microbiota (bacteria, viruses, and fungi) and the regulation of the intestinal immune system, which predisposes individuals to the risk of both UC and CD.^23^ Although the role of bacteria in this context is relatively well-established, studies have focused more on bacterial effects rather than those of viruses, protists, and fungi.^26,27^ Some studies conducted on patients with UC have revealed a decrease in bacterial diversity in the gut, particularly a decrease in Firmicutes and an increase in Gammaproteobacteria and Enterobacteriaceae.^28^ However, despite extensive research, it is still not clear whether changes in the quantity and nature of bacteria are the cause or the consequence of mucosal inflammation.

## Results

 Several studies have demonstrated that dietary factors can be associated with the pathogenesis and progression of UC by directly influencing the composition or function of the intestinal microbiota. Diet plays a significant role in shaping the composition of the intestinal microbiota. For instance, an increase in the abundance of Bacteroides has been found to be important in shaping the microbial composition of the intestine.^29^ In line with this, a study comparing African and European children found that the decrease in Firmicutes and Enterobacteriaceae in African children was primarily attributable to differences in dietary patterns between the two populations.^30^ Therefore, it is suggested that diet-induced changes in the microbiota may transform a healthy gut into a pathogenic environment that can initiate or perpetuate inflammation in the intestine.^29^ However, it is important to note that establishing a definitive cause-and-effect relationship between the microbiome and the outcomes of IBD is challenging, and most studies have utilized cross-sectional and longitudinal designs that may have limitations in demonstrating causation.^31^

 Animal studies have provided further insights into the impact of a high-fat and low-fiber diet on gut microbial composition and the initiation of IBD. These studies have shown that such a diet can accelerate the development of IBD in mice, suggesting a correlation between a high-fat and low-fiber diet and the incidence of IBD.^32^ Epidemiological studies have also explored the relationship between dietary habits and the risk of IBD. For example, a study conducted within the UK Biobank, involving 121 490 participants, found a correlation between the consumption of sugar-sweetened beverages (rather than artificially sweetened beverages or natural juices) and the risk of IBD.^33^ Another prospective cohort study conducted in the United States, involving 245 112 participants, revealed that a higher intake of ultra-processed foods was associated with an increased risk of CD incidence.^34^ However, not all studies have demonstrated significant associations between dietary habits and the incidence of IBD. A cohort study in France, which included a total of 105,832 participants with different dietary habits, found no association between these habits and the incidence of IBD.^35^ Furthermore, inadequate fiber intake has been observed in patients with IBD, highlighting the importance of proper fiber consumption in disease management.^36^

 In addition to influencing the composition of the gut microbiota, nutrients can also affect microbial metabolism. Short-chain fatty acids (SCFAs), produced through bacterial fermentation of indigestible fibers, play a crucial role in maintaining the integrity of the intestinal mucosal barrier and regulating immune function.^37-39^ Studies evaluating patients with IBD have shown a decrease in SCFA levels associated with a Western dietary pattern.^40^ Animal studies focusing on the balance of macrobiota have indicated that dysregulation of the intestinal microbiota composition caused by a Western diet can lead to the overgrowth of pro-inflammatory proteobacteria, including *E. coli*, which subsequently compromises the protective bacterial barrier of the intestinal wall and contributes to the development of IBD.^40^

 It is important to note that a Western diet encompasses not only the nutritional components but also includes industrial artificial additives such as food preservatives, food additives, sweeteners, and processed foods. Some studies have suggested a possible correlation between these factors and the incidence of IBD.^16,17^ However, it should be emphasized that there is currently insufficient human experimental evidence to establish a definitive link between these factors and IBD.

 Overall, the relationship between diet and IBD is complex and involves various factors, including the composition of the gut microbiota, immune responses, and the integrity of the intestinal barrier. While a Western diet high in fat and sugar and low in fiber has been associated with an increased risk of IBD, more research is needed to fully understand the underlying mechanisms and to determine the specific dietary interventions that may help prevent or manage IBD effectively.

## Carbohydrate

 Carbohydrate consumption has been associated with signs and symptoms of IBD. When bacteria digest non-absorbed carbohydrates in the gut, it can lead to bacterial overgrowth and the production of harmful chemicals that cause intestinal inflammation.^41^

 Two dietary approaches restricting carbohydrate consumption are the specific carbohydrate diet (SCD) and the Fermentable Oligosaccharides, Disaccharides, Monosaccharides, and Polyols (FODMAPs) diet. The FODMAP diet, which restricts certain types of carbohydrates that are poorly absorbed, has been suggested to increase the osmotic gap and luminal water in the intestines, leading to inflammation and exacerbation of IBD symptoms.^42^ Some studies have shown that a low-FODMAP diet can reduce gas production, abdominal distention, and pain in patients with IBD.^41,42^ However, a randomized clinical trial found no significant changes in inflammation markers after a low-FODMAP diet intervention, although there were slight alterations in gut microbiota.^43^ Another trial reported that a low-FODMAP diet led to a slight increase in the percentage of patients with IBD and inactive disease, improvements in disease activity, and decreased fecal biomarkers of intestinal inflammation.^44^ However, it should be noted that the long-term effects and efficacy of the low-FODMAP diet in IBD are still not well-established.

 The SCD, introduced in the 1920s, is a diet low in carbohydrates except for monosaccharides. It is believed to affect the composition of the intestinal microbiome and reduce intestinal inflammation.^41^ Limited data are available on the efficacy of the SCD, particularly in adults. Some studies have reported improvements in clinical symptoms for patients on the SCD diet, but long-term compliance can be challenging.^45,46^ A randomized trial comparing the SCD with the Mediterranean diet did not find significant symptomatic relief in patients with IBD following the SCD.^47^ Case studies have shown mixed results, with some patients showing improvements in symptoms but no progress in mucosal healing, while others have shown improvements in endoscopic, histologic, and laboratory markers.^48,49^ Nonetheless, another case study on a male patient with moderate-to-severe CD reported that after 42 months of adhering to SCD diet, the endoscopic, histological, and laboratory markers improved, and the patient showed weight loss.^17,50^

 Overall, carbohydrate consumption, especially certain types of carbohydrates, may have an impact on IBD symptoms and intestinal inflammation. The FODMAP diet and the SCD are two dietary approaches that restrict carbohydrate intake, but further research is needed to fully understand their efficacy and long-term effects in the management of IBD. It is important for individuals with IBD to work with healthcare professionals to determine the most appropriate dietary approach for their specific condition.

## Gluten and Other Participating Factors

 Gluten, found in wheat, barley, and rye, is known to trigger celiac disease, but a gluten-free diet (GFD) is not recommended for patients with IBD according to recent guidelines.^51^ However, the potential role of a GFD in IBD pathogenesis has been investigated in several studies, and it has been found that a significant number of patients with IBD follow a GFD.^17,52^

 In addition to gluten, modern dietary habits, and processed foods have been suggested to increase the incidence of IBD. A prospective cohort study found that ultra-processed foods such as processed meats, soft drinks, refined sweetened foods, salty foods, and snacks were associated with a higher prevalence of IBD.^52^ The study proposed that the food processing method, rather than the specific food items, might contribute to the increased risk of IBD.^52^ However, another cohort study did not find a significant association between IBD and ultra-processed food intake.^53^

 Other precipitating factors in IBD include specific dietary items. A cohort study found that individuals with active IBD consumed more alcohol, popcorn, nuts, legumes, deep-fried food, processed deli meat, seeds, sports drinks, and sweetened beverages compared to those with inactive disease.^35^ A plant-based diet has shown potential benefits in IBD management, with studies reporting lower relapse rates and clinical remission in patients following a plant-based diet.^54,55^ Additionally, the consumption of extra virgin olive oil has been associated with improvements in symptoms and inflammatory markers in patients with UC.^56^ A modified Mediterranean diet, including certain amounts of fruit, vegetables, olive oil, and fish, has been reported to be irrelevant to the risk of older-onset UC, while the risk of developing CD later in life was reduced by adhering more closely to a modified Mediterranean diet.^48^ Table 1 provides a summary of the general characteristics of the included studies.

**Table 1:** Summary of the evaluated studies

**First author**	**Country**	**Study year**	**Study design**	**subject**	**Outcome**
Kuda et al^32^	Japan	2017	Clinical trial On mice	effect of a high-fat and low-fiber diet on the gut microbial composition and initiation of IBD	Positive correlation
Fu et al^33^	England	2022	cohort study	Sweetened beverages or natural juices and IBD risk	Positive correlation
Lo et al^34^	United States	2021	Nation-wide prospective cohort study	UPF intake and risk of CD incidence	Positive correlation
Vasseur et al^35^	France	2020	cohort study	“Healthy”, “traditional,” and “Western” Dietary habits and incidence of IBD	Negative correlation
Davis et al^36^	-	2020	Multicenter cross-sectional study	Habitual dietary fiber intake and risk of IBD	Positive correlation
Agus et al^40^	France	2016	Randomized clinical trial	the effect of Western diet and consequently adherent-invasive *E. coli* in aggravation of IBD	Significant correlation
Cox et al^43^	England	2019	randomized clinical trial	The effect of low-FODMAP on the inflammatory status of gut	Negative correlation
Bodini et al^44^	Italy	2019	randomized clinical trial	Correlation between FODMAP diet and activation of IBD in patients	Positive correlation
Cox et al^57^	England	2021	case-control	Correlation between FODMAP diet and activation of IBD in patient	Positive correlation
Lewis et al^47^	United States	2021	randomized trial	the effects of SCD and the Mediterranean diet on the symptoms of IBD patients	Negative correlation
Britto et al^49^	United States	2020	Case series	The effect of SCD diet on the symptoms of IBD patients	Positive correlation
Arjomand et al^50^	United States	2022	Case report	The effect of SCD diet on the symptoms of IBD patients	Positive correlation
Narula et al^53^	Canada	2021	prospective cohort study	The relationship between ultra-processed food and IBD incidence	Positive correlation
Vasseur et al^35^	France	2020	Cohort study	Association between IBD and ultra-processed food intake	Negative correlation
Khalili et al^48^	Sweden	2022	Prospective cohort study	relationship between diet quality and risk of older-onset IBD	Negative correlation with UC, Positive correlation with CD
Vagianos et al^54^	Canada	2014	cohort study	Evaluation of dietary habits in IBD patients	Positive correlation
Chiba et al^55^	Japan	2019	single-group trial study	Relationship between plant-based diet and relapse in IBD patients	Positive correlation
Morvaridi et al^58^	Iran	2020	clinical trial	The effect of olive oil on symptom remission in UC patients	Positive correlation

## Conclusion

 In conclusion, dietary habits play a significant role in the incidence and management of IBD. Dysregulations of cell wall permeability and immune response are major factors in IBD, and adopting a controlled dietary habit can potentially help control the occurrence of IBD. However, it is important to note that individual responses to dietary changes may vary, and it is advisable for patients with IBD to work with healthcare professionals to determine the most appropriate dietary approach for their specific condition. Therefore, it is reasonable that gastroenterologists evaluate the dietary habits of the patients and the patient’s family and advise them to reduce the consumption of a predisposing diet in order to lessen the likelihood of flairs and also to ameliorate the risk for other family members.
